# Novel Nuclease MbovP701 with a Yqaj Domain Is Interrelated with the Growth of *Mycoplasma bovis*

**DOI:** 10.3390/microorganisms12122509

**Published:** 2024-12-05

**Authors:** Zhiyu Hao, Doukun Lu, Xixi Li, Abdul Raheem, Gang Zhao, Ali Sobhy Dawood, Yingyu Chen, Xi Chen, Changmin Hu, Jianguo Chen, Lei Zhang, Xifang Zhu, Aizhen Guo

**Affiliations:** 1 National Key Laboratory of Agricultural Microbiology, Huazhong Agricultural University, Wuhan 430070, China; 2 Hubei Hongshan Laboratory, Huazhong Agricultural University, Wuhan 430070, China; 3 College of Veterinary Medicine, Huazhong Agricultural University, Wuhan 430070, China; 4 Hubei International Scientific and Technological Cooperation Base of Veterinary Epidemiology, Huazhong Agricultural University, Wuhan 430070, China; 5 International Research Center for Animal Disease, Ministry of Science and Technology, Huazhong Agricultural University, Wuhan 430070, China; 6 Key Laboratory of Ministry of Education for Conservation and Utilization of Special Biological Resources in the Western China, School of Life Sciences, Ningxia University, Yinchuan 750021, China; 7 Department of Medicine and Infectious Diseases, Faculty of Veterinary Medicine, University of Sadat City, El Sadat City 32897, Egypt; 8 School of Life Sciences, Zhengzhou University, Zhengzhou 450001, China

**Keywords:** *Mycoplasma bovis*, MbovP701, nuclease, YqaJ domain, growth deficiency, bovine lung epithelial cells

## Abstract

*Mycoplasma bovis* (*M. bovis*) is characterized by a reduced genomic size and limited synthetic capacity, including the inability to synthesize nucleotides de novo, relies on nucleases for nutrient acquisition and survival. A number of nucleases have been implicated in *M. bovis* pathogenicity, facilitating substrate degradation and contributing to DNA repair mechanisms that enhance bacterial persistence. The present study confirmed that the T5.808 mutant, in which a novel nuclease gene (Mbov_0701) was disrupted by the mini-Tn4001 transposon, exhibits a growth defect when co-cultured with EBL cells. However, the restoration of Mbov_0701 resulted in the resumption of growth in the mutant. The characterization of MbovP701 revealed that it had high activity in hydrolyzing dsDNA with 5′- to 3′- polarity. Furthermore, the substrates of MbovP701 were extended to include linear dsDNA, ssDNA, RNA, and plasmid DNA. The exonuclease activity is dependent on the presence of Mn^2+^ and/or Mg^2+^ ions, with an optimal pH and temperature of 8.3 and 43 °C, respectively. The truncation experiments of rMbovP701 revealed that YqaJ (41–185 aa) is the key functional domain of MbovP701 exonuclease. In conclusion, the present study identified a novel nuclease in *M. bovis* that plays an essential role in the proliferation of this minimal organism. This finding elucidates the survival strategy and pathogenesis of *M. bovis*, suggesting a potential therapeutic strategy for the treatment of *M. bovis* through targeting the inhibition of MbovP701. Moreover, it provides a foundation for future investigations into the interactions between MbovP701 and other nucleases involved in *M. bovis* biology.

## 1. Introduction

*Mycoplasma bovis* (*M. bovis*) is an important causative agent of the bovine respiratory disease complex (BRD), mastitis, arthritis, keratoconjunctivitis, and other diseases [1]. It was first reported as a cause of bovine mastitis in the United States in 1961, and then identified as a major cause of respiratory disease in cattle in 1976 [2]. Due to their minimal genome and limited biosynthetic capabilities, mycoplasmas rely heavily on colonization and the acquisition of host-derived nutrients to sustain survival, replication, and virulence. Previous studies have elucidated several mechanisms underlying the pathogenesis of *M. bovis* [3]. However, research on the interactions between mycoplasmas and their hosts has faced considerable challenges, primarily due to the limited availability of tools for manipulating mycoplasma genomes and the absence of suitable animal models for disease modeling [4].

Nucleases are critical virulence factors in mycoplasma infections, as these bacteria are unable to synthesize nucleotides de novo and must degrade environmental nucleic acids to acquire the necessary nucleotides for their metabolism [5]. Recent studies have identified numerous nucleases in mycoplasma species, many of which contain *Staphylococcus aureus* nuclease (SNc) domains. These nucleases contribute to host pathogenicity and cytotoxicity by degrading nucleotides and inducing apoptosis-like cell death mechanisms during infection [6,7,8,9,10]. In addition, three phosphodiesterase (PDE) proteins endowed with DHH domains have been identified within *M. bovis*. Notably, MbovP327 and MbovP328 were observed to exhibit activity towards nanoRNAs, whereas MbovP276 (MbovGdpP) was shown to have both nanoRNase and single-stranded DNase activities [11,12]. Importantly, these enzymes were able to convert their substrates into mononucleotides, and all of these enzymes are critical for the growth and survival of *M. bovis*.

Lambda exonuclease (Lambda-Exo) is a member of the type 2 restriction endonuclease (T2RE) family of nuclease enzymes that share the conserved PD-(D/E)XK motif [13,14]. This extensive family encompasses numerous restriction enzymes along with others essential for DNA repair and RNA processing. Prior to 1967, a 226-amino acid Lambda-Exo was discovered in an *E. coli* Lambda phage that was capable of cleaving linear dsDNA from 5′ to 3′ ends, resulting in the release of 5′ monodeoxyribonucleic acid and the production of long 3′ ssDNA ends [15,16]. The high-resolution crystal structure of Lambda-Exo reveals a unique ring-shaped homotrimeric architecture with a central channel that serves as a conduit for the sequential tracking and processing of the DNA substrate [17]. Furthermore, Lambda-Exo plays a central role in recombination events occurring within the DNA of bacteriophage lambda [18,19]. As research has progressed, Lambda-Exo-like proteins have been found in various bacteria and viruses, with examples being the SOX protein of Kaposi’s sarcoma-associated herpesvirus (KSHV) and the SXT-Exo protein of *Vibrio cholerae* [20,21,22]. In this context, it is worth exploring the role of Lambda-Exo-like proteins, which are found in various bacteria and viruses but have yet to be reported in mycoplasma species. A comprehension of the functional parallels between Lambda-Exo and nuclease activity in *M. bovis* may facilitate an appreciation of the evolutionary and mechanistic foundations of nuclease-mediated pathogenesis in this minimal bacterium.

In our previous study, we screened 13 mutants with a growth defect phenotype in cell co-culture, one of which carried a mutation in the Mbov_0701 gene [11]. This finding suggested the involvement of the Mbov_0701 gene and highlighted its important role in the growth and metabolism of *M. bovis*. Furthermore, bioinformatic prediction revealed that the MbovP701 protein is a nuclease containing the YqaJ domain found in Lambda-Exo. Therefore, this study aimed to characterize the nuclease activity associated with this domain and its effect on growth in PPLO medium and co-culture conditions with fetal bovine lung epithelial (EBL) cells. The results would contribute to the elucidation of the survival strategy and pathogenesis of *M. bovis* and inform the development of effective control measures against *M. bovis*-associated diseases.

## 2. Materials and Methods

### 2.1. Ethics Statement

Animal experiments were conducted in strict accordance with the *Guide for the Care and Use of Laboratory Animals*, Monitoring Committee of Hubei Province, China, and protocols were approved by the Committee on the Ethics of Animal Experiments at the College of Veterinary Medicine, Huazhong Agricultural University (agreement no. SYXK(ER) 2015-0084 issued on 31 October 2015). To ensure animal welfare, the animals were housed in standardized facilities and provided with nutritious feed and clean water. Regular health checks were performed, and the animals were monitored daily for signs of distress, illness, or abnormal behavior. Any necessary interventions were promptly administered. Pain management adhered to the “3Rs” principles, disturbances were minimized, and proper post-experimental disposal procedures were followed.

### 2.2. Bacterial Strains and Cell Culture Conditions

The *M. bovis* HB0801 strain used in this study is maintained at the China Center for Type Culture Collection (CCTCC no: M2010040), Wuhan. It was isolated from Hubei, China, in 2008 at our laboratory. The PPLO medium has been specifically designed for the cultivation of mycoplasma species due to its optimized nutrient composition, including pyruvate and horse serum, which are essential for the growth of *M. bovis*. Accordingly, the mycoplasma was grown in PPLO (BD Company, Sparks, MD, USA) as previously described [23]. *M. bovis* HB0801 mutant strains T5.808 and its complementary CT5.808 were grown in PPLO medium with 100 μg/mL gentamicin or 10 μg/mL puromycin, respectively.

The embryonic bovine lung (EBL) cell line, generously donated by Prof. Fei Xue, was grown in minimum essential medium (MEM) supplemented with 10% heat-inactivated fetal calf serum (Gibco, Grand Island, NY, USA) at 37 °C under an atmosphere of 5% CO_2_/95% air.

### 2.3. The Construction of the CPT5.808 Strain Complementing the Mbov_0701 Mutant

The Mbov_0701-knockout mutant T5.808 was identified from a transposon-mediated *M. bovis* mutant library previously constructed in this laboratory [11]. To generate the complement strain, the DNA fragment of Mbov_0701 under the control of the P40 promoter was synthesized by Beijing Tianyi Huiyuan Bioscience & Technology Inc and ligated into plasmid pOH/P at the site of *Not*I to obtain recombinant plasmid pOH/P-Mbov_0701. Competent T5.808 cells were transfected with pOH/P-Mbov_0701 to generate the complementing CPT5.508 strain, while HB0801 cells were transfected with the pOH/P vector as the control using previously described methods [24]. Briefly, T5.808 was cultivated until the late log phase, then subjected to centrifugation at 10,000 rpm for 10 min at room temperature, followed by three washes with DPBS. The pellet was resuspended with 375 μL of 0.1 M CaCl_2_. Subsequently, 3 μg of pOH/P-Mbov_0701 plasmid and 1 µL of yeast tRNA were mixed and added to 100 µL of competent cells. The mixtures were then transferred into 1 mL of PEG8000 (Sigma–Aldrich Corporation, St. Louis, MO, USA). After a 1 min incubation, 5 mL of PPLO medium was added and incubated at 37 °C for 3 h. The cultures were collected and resuspended in 1 mL of PPLO-selective medium containing 10 μg/mL puromycin. After overnight incubation, *M. bovis* was inoculated on selective agar plates and single colonies were picked and stored at −80 °C.

The expression of the MbovP701 in the mutant T5.808 strain and its complementary strain CPT5.808 was verified through a Western blotting assay. The wild-type strain HB0801, mutant T5.808, and complement CPT5.808 strains were cultured in 1 mL of PPLO medium with the necessary antibiotics for 36 h and precipitated by centrifugation at 12,000× *g* for 5 min. The pellets were suspended using 100 μL PBS and 20 μL of 5× protein loading buffer, and then boiled for 10 min. The protein was subjected to SDS-PAGE and transferred onto a PVDF membrane (Millipore, Bedford, MA, USA). Rabbit antiserum (1:500) raised against MbovP701 was employed as the primary antibody, while mouse antiserum MbovP579 (1:500) was used as the control. Horseradish peroxidase (HRP)-conjugated goat anti-rabbit IgG antibody and goat anti-mouse IgG antibody (1:5000; Southern Biotech, AL, USA) were used as secondary antibodies. Protein bands were detected using WesternBright™ ECL (Advansta, Menlo Park, CA, USA).

### 2.4. Growth Curve and Colony Morphology of T5.808 Mutant, CPT5.808, and Wild-Type Strain

The *M. bovis* wild-type HB0801, T5.808, and CPT5.808 strains were diluted into 10^5^ CFU/mL in PPLO medium, and inoculated into PPLO medium at a ratio of 1:10. The samples were incubated continuously for 72 h in an incubator with 5% CO_2_ at 37 °C, and the appropriate bacterial solution was taken every 12 h for colony count. *M. bovis* (HB0801, T5.808, CPT5.808) and EBL cells were co-cultivated in MEM supplemented with 2 mM L-glutamine and Earle’s balanced salts. EBL cells were seeded at a density of 2 × 10^4^ cells/cm^2^ in 24-well plates and infected with *M. bovis* at the multiplicity of infection (MOI) of 0.5. *M. bovis* and the EBL cells were allowed to grow at 37 °C under an atmosphere of 5% CO_2_/95% air. At different times post inoculation (24, 48, and 72 h), *M. bovis* titers were determined by CFU titrations following one freeze–thaw (−80 °C/+37 °C) cycle to release intracellular bacteria. The generation time of *M. bovis* in both cell co-culture conditions and axenic growth during the exponential phase was calculated in accordance with the formula described previously [25].

### 2.5. Bioinformatics Analysis

The Mbov_0701 sequence (old locus tag as “Mbov_RS03450”) of *M. bovis* HB0801 genome (accession number: WP_013456606.1) was retrieved from the NCBI database and analyzed using the online PROSITE database. Basic gene features, such as molecular weight and isoelectric point, etc., were predicted using the online PROSITE database https://web.expasy.org/protparam/, accessed on 9 September 2020. Conserved domains within the gene sequence were analyzed using the Conserved Domains tool available on the NCBI database. To determine whether MbovP701 is a secreted protein, signal peptide sequences and cleavage sites were predicted using Signal IP (http://www.cbs.dtu.dk/services/SignalP/, accessed on 9 September 2020), and transmembrane regions were identified using TMHMM2.0 (http://www.cbs.dtu.dk/cgi-bin/webface2.Fcgi?Jobid=60b7099c000071e12478c0df&wait=20, accessed on 9 September 2020).

### 2.6. Expression and Purification of rMbovP701 and Truncated Protein

The Mbov_0701 gene was cloned from the *M. bovis* HB0801 genome using overlapping PCR. To overcome the tryptophan codons barrier (UGA) in translating *M. bovis* genes in *E. coli*, site-directed mutagenesis was employed to mutate the TGA codon to TGG using primers 0701-F1/0701-R1, 0701-F2/0701-R2, and 0701-F3/0701-R3 (Table S1). The truncated versions of Mbov_0701 corresponding to proteins MbovP701^Δ186–296^, MbovP701^Δ1–40^, MbovP701^Δ41–185^, and MbovP701^Δ1–40, 186–296^ were synthesized by Beijing Tianyi Huiyuan Bioscience & Technology Inc. Each of these fragments was ligated into pET-30a (+) vector and then transferred into *E. coli* BL21 (DE3) for protein expression. Briefly, *E. coli* was grown in LB medium containing 50 μg/mL kanamycin at 37 °C for 3 h. Subsequently, 0.8 mM isopropyl-β D-thiogalactose (IPTG) was added to the culture. After continued cultivation for 3–4 h, the cultures were collected and resuspended in binding buffer (2 mM imidazole, 20 mM Na_3_PO_4_, 500 mM NaCl; pH 7.4). The cultures were then homogenized at 1000 bar (4 °C) 3–4 times, and soluble proteins were collected by centrifugation 12,000× *g* for 30 min before loading onto nickel affinity chromatography (GE Healthcare, Piscataway, NJ, USA). The purified proteins were analyzed by SDS-PAGE and quantified using the BCA protein assay (Thermo Fisher Scientific, Waltham, MA, USA), respectively.

### 2.7. Preparation of Rabbit Anti-rMbovP701 Polyclonal Antibody

The rMbovP701 antiserum was produced by immunizing New Zealand rabbits at 6 weeks of age. The rabbits were initially primed with 200 μg of rMbovP701 mixed with an equal volume of Freund’s complete adjuvant (Sigma–Aldrich Corporation, St. Louis, MO, USA) and subsequently boosted twice with the same doses of protein and Freund’s incomplete adjuvant at two-week intervals. After the last immunization, the rabbits were euthanized, bled, and the serum samples were collected and used for immunological characterization.

### 2.8. Analysis of Nuclease Activity

To confirm the digestive polarity of rMbovP701, two 712 bp dsDNA substrates—one unmodified and the other phosphorothioate-modified-were amplified by PCR with pET-32a as a template, with primers listed in Table S1. Subsequently, the two dsDNA substrates (1 μg) were separately incubated with 2.5 μg of Lambda-Exo (NEB, Beijing, China) or rMbovP701 in Tris-HCl buffer (25 mM, pH8.0), containing 7.5 mM MgCl_2_ and 1 mM DTT at 37 °C. After 1 h, thee reaction mixture was quenched immediately using 10 mM EDTA, followed by analysis on 1% agarose TAE gels.

Nuclease activity of rMbovP701 was analyzed as described [8]. Briefly, 2.5 μg of rMbovP701 was incubated at 37 °C in 50 µL reaction buffer (50 mM Tris-HCl, pH8.0; 50 mM NaCl; and 7.5 mM MgCl_2_) containing 1 μg of ssDNA (Table S1), dsDNA (Table S1), total RNA from Mac-T cells, or pET-30a (+) plasmid. In total, 50 μL of the reaction mixture was withdrawn at various time points (0, 10, 20, 30, 60, and 120 min), and the reaction was terminated by the addition of 10 mM EDTA. Subsequently, the reaction products were subjected to analysis via 1% agarose/TAE gel. To determine the optimal metal ion concentration, 2.5 μg rMbovP701 was co-incubated with different concentrations of metal ions including MgCl_2_ (0.1–100 mM), MnCl_2_ (0.1–20 mM), KCl (0.1–100 mM), NaCl (0.1–10 mM) and CaCl_2_ (0.1–100 mM). Subsequently, 1 μg dsDNA was added, and the mixture was incubated for 20 min. The optimal pH and temperature conditions were investigated by adjusting the reaction buffer (25 mM Tris-HCl, 7.5 mM MgCl_2_) to a series of pH values (pH 7.0–9.0) and temperature ranges (30–72 °C). Picogreen™ quantitative reagent (Thermo Fisher Scientific, Waltham, MA, USA) was utilized to analyze the optimal enzymatic activity conditions. Additionally, the nuclease activities of rMbovP701’s truncated proteins were evaluated under optimal reaction conditions.

### 2.9. Statistical Analyses

A one-way ANOVA was conducted for multiple comparisons using GraphPad Prism 8.0 software. Significant differences were identified as *p* * < 0.05, *p* ** < 0.01, *p* *** < 0.001 and “ns” represented non-significance.

## 3. Results

### 3.1. MbovP701 Is Crucial for M. bovis Growth

**Figure 1:**
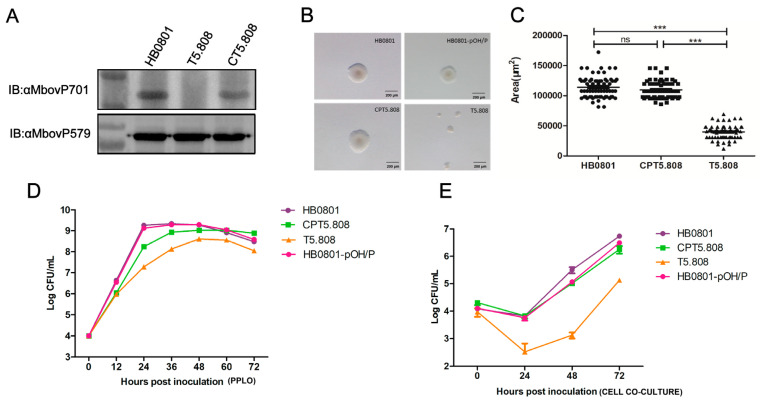
The colony morphology and growth characteristics of *M. bovis.* (**A**) A visualization of MbovP701 expression in HB0801, T5.808, and CPT5.808 with a Western blotting assay; MbovP579 was used as the internal control. (**B**) The colony morphology of HB0801, HB0801-pOH/P, T5.808, and CPT5.808 on PPLO agar plate; each colony was magnified 30 times under a microscope. (**C**) The colony size of HB0801, T5.808, and CPT5.808; 65 colonies of each strain were randomly measured; *** *p* < 0.001, ns means no significant difference. (**D**) The growth curve of HB0801, HB0801-pOH/P, T5.808, and CPT5.808 in PPLO medium. (**E**) The growth curve of HB0801, HB0801-pOH/P, T5.808, and CPT5.808 under EBL cell culture conditions. Data means are representative of three independent experiments, and the error bar indicates SD.

To ascertain the influence of MbovP701 on the growth and metabolic processes of *M. bovis*, we first verified the expression of MbovP701 in the mutant strain and complementary strain. The results revealed that MbovP701 was not expressed in the T5.808 mutant, whereas restoration of expression was observed in its complementary strain, CPT5.808 (Figure 1A). Colony morphology analysis showed a significant reduction in colony size for the mutant strain T5.808 compared to the wild-type strain HB0801 and the complementary strain CPT5.808 (Figure 1B,C).

Additionally, the growth curves of *M. bovis* in PPLO medium revealed delayed growth and a reduction in final titers (approximately 10^8^ CFU/mL) for the mutant T5.808 in comparison to the HB0801 and empty plasmid transfection HB0801-pOH/P strains. The generation time of T5.808 was calculated to be 2.20 h ± 0.011, while the generation times of HB0801 and HB0801-pOH/P were 1.37 h ± 0.008 and 1.39 h ± 0.024, respectively. Meanwhile, the complementary strain CPT5.808 exhibited a partial restoration of growth (Figure 1D), with a generation time of 1.71 h ± 0.015. In co-culture with EBL cells, the strains HB0801, HB0801-pOH/P, and CPT5.808 displayed substantial growth, with a generation time of approximately 2.48 h ± 0.21, 2.64 h ± 0.12, and 2.97 h ± 0.23, respectively. Furthermore, the titer reached 10^6^ CFU/mL after 72 h. In contrast, the growth of T5.808 was significantly slower than that of the other strains, reaching only 10^5^ CFU/mL after 72 h (Figure 1E), with a generation time of 3.40 h ± 0.07. These findings provide evidence that the Mbov_0701 gene is essential for the growth of *M. bovis* under both PPLO medium and cell culture conditions.

### 3.2. The YqaJ Domain MbovP701 Exhibited Exonuclease Activity from a 5′ to 3′ Direction

**Figure 2:**
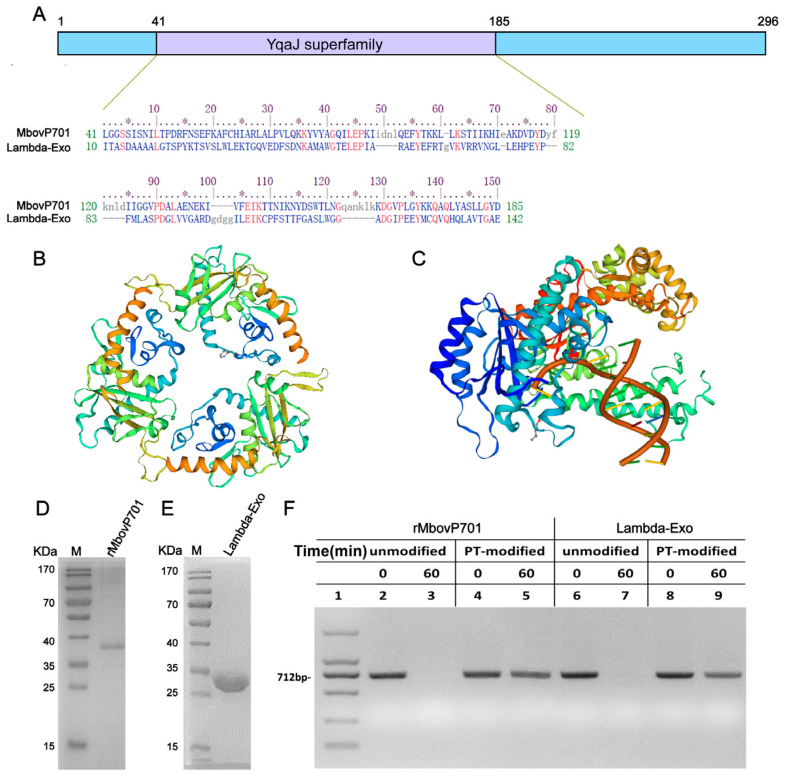
The sequence analysis and nuclease degradation direction of rMbovP701. (**A**) The sequence alignment and structural modeling of the MbovP701 protein. The protein contains 296 amino acids, and this region (41–185) is very similar to the functional domain of Lambda-Exo; (**B**) a ribbon diagram of the MbovP0701 model with structural features, showing the ring homo-trimer structure of the protein; (**C**) the MbovP0701 toroid-bound dsDNA pattern diagram, showing a ring-shaped toroid with a central tapered channel. The inner diameter of the wide end of the channel is spacious enough to accommodate dsDNA, whereas the inner diameter of the narrow end of the channel is only capable of accommodating ssDNA; (**D**,**E**) SDS-PAGE staining of purified rMbovP701 and Lambda-Exo nucleases by Coomassie bright blue; (**F**) the degradation of two different modified substrates by rMbovP701 and Lambda-Exo; M (kDa). The marker represents protein standards of different molecular masses.

MbovP701 is a 34.76 kDa protein comprising 296 amino acids, with an isoelectric point (pI) of 8.13. The amino acid sequence lacks both a signal peptide (Sec/SPI score: 0.0003) and a transmembrane region. The YqaJ-like viral recombinase functional domain thought to be responsible for cleaving nucleotides is located between the 41- and 185-amino acid region within MbovP701 (Figure 2A). Secondary structure predictions indicate the presence of α-helix structures in multiple regions of MbovP701, while tertiary structure modeling suggests that MbovP701 shares a spatial configuration similar to Lambda-Exo. The predicted structure forms a ring-shaped homotrimer with a cone-shaped central channel capable of accommodating the passage of dsDNA (Figure 2B,C).

Previous studies have shown that Lambda-Exo degrades linear double-stranded DNA in a 5′ to 3′ direction [16,26]. Given the high degree of homology between the YqaJ domain of MbovP701 and Lambda-Exo, we hypothesize that MbovP701 exhibits analogous functionality. Thus, the rMbovP701 proteins, approximately 37 kDa, were successfully purified (Figure 2D). The 5′-terminal phosphorylated dsDNA (PT-modified) and unmodified dsDNA were incubated with the rMbovP701 or Lambda-Exo protein, respectively. The results showed comparable degradation patterns for rMbovP701 and Lambda-Exo, both of which were capable of degrading unmodified dsDNA substrates. However, neither enzyme could degrade thiophosphorylated substrates (Figure 2E,F). These findings substantiate the hypothesis that rMbovP701 functions as a 5′-3′ exonuclease to degrade dsDNA.

### 3.3. rMbovP701 Is a Broad-Spectrum Exonuclease

**Figure 3:**
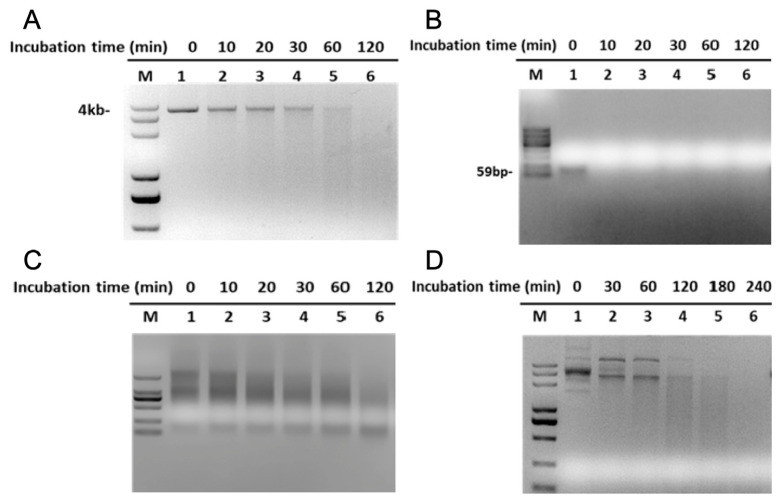
An assay of rMbovP701 nuclease activity. (**A**) The dsDNA digested by rMbovP701; (**B**) the ssDNA digested by rMbovP701; (**C**) the MAC-T cellular RNA digested by rMbovP701; (**D**) the plasmid DNA of pET-30a (+) digested by rMbovP701; M: DNA marker.

The above data illustrate that rMbovP701 is capable of degrading linear dsDNA. To ascertain the substrate specificity of MbovP701, we employed a series of nucleic acid substrates, including dsDNA, ssDNA, RNA, and cyclic plasmids. As the reaction progressed, rMbovP701 exhibited degradation activity towards all tested substrates, including dsDNA, ssDNA, RNA, and cyclic plasmid DNA (Figure 3). It is noteworthy that the degradation of ssDNA is most prominent, with the substrate being completely degraded within 10 min (Figure 3B). Furthermore, the degradation of dsDNA and RNA was clearly evident, with the appearance of diffuse bands occurring approximately 60 min following the initiation of the reaction (Figure 3A,C). The cyclic plasmid DNA exhibited varied states, transitioning from supercoiled to the open-loop and linear forms following the reaction, and gradually disappearing with prolonged incubation time (Figure 3D). Taken together, these results demonstrate that rMbovP701 has multifunctional exonuclease activity.

### 3.4. rMbovP701 Is a Mg^2+^/Mn^2+^-Dependent Thermostable Alkaline Exonuclease

**Figure 4:**
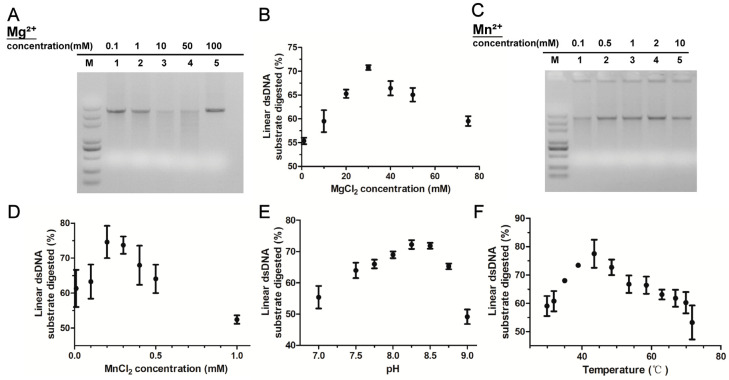
The effect on rMbovP701 activity by different metal ions, pH, and temperature. (**A**–**D**) The degradation of dsDNA incubated with rMbovP701 in the presence of different concentrations of MgCl_2_ (**A**,**B**) and MnCl_2_ (**C**,**D**). (**E**,**F**) The degradation of dsDNA by rMbovP701 under various pH (**E**) and temperature (**F**) conditions. Data are representative of three independent experiments; the error bar indicates standard deviation (SD).

We predicted that MbovP701 functions as a Mg^2+^-dependent exonuclease. Nucleic acid electrophoresis analysis revealed the significant degradation activity of MbovP701 protein toward dsDNA in the presence of Mg^2+^ concentrations ranging from 10 to 50 mmol/L, indicating that this range represents the optimal concentration (Figure 4A). Additionally, parallel comparisons were conducted to assess the degradation activity of rMbovP701 in the presence of Mn^2+^, K⁺, Na⁺, and Ca^2+^ ions. Notably, under different concentrations of Mn^2+^, only a low concentration of 0.1 mmol/L exhibited a degradation effect (Figure 4C). Partial degradation of dsDNA was observed at 100 mmol/L concentrations of K⁺ and Na⁺, whereas degradation in the presence of Ca^2+^ was negligible (Figure S1). Subsequently, the Picogreen^TM^ dsDNA quantitative reagent was used to monitor the digestion of dsDNA by the nuclease over time under Mg^2+^ and Mn^2+^, revealing the strongest degradation activity at concentrations of approximately 30 mmol/L Mg^2+^ (Figure 4B) and 0.2 mmol/L Mn^2+^ (Figure 4D). Moreover, rMbovP701 was shown to function as a thermostable alkaline exonuclease, with optimal enzymatic activity at pH 8.3 (Figure 4E) and a suitable reaction temperature of 43 °C for optimal enzymatic activity (Figure 4F).

### 3.5. The YqaJ Domain of rMbovP701 Is Necessary for Its Exonuclease Activity

**Figure 5:**
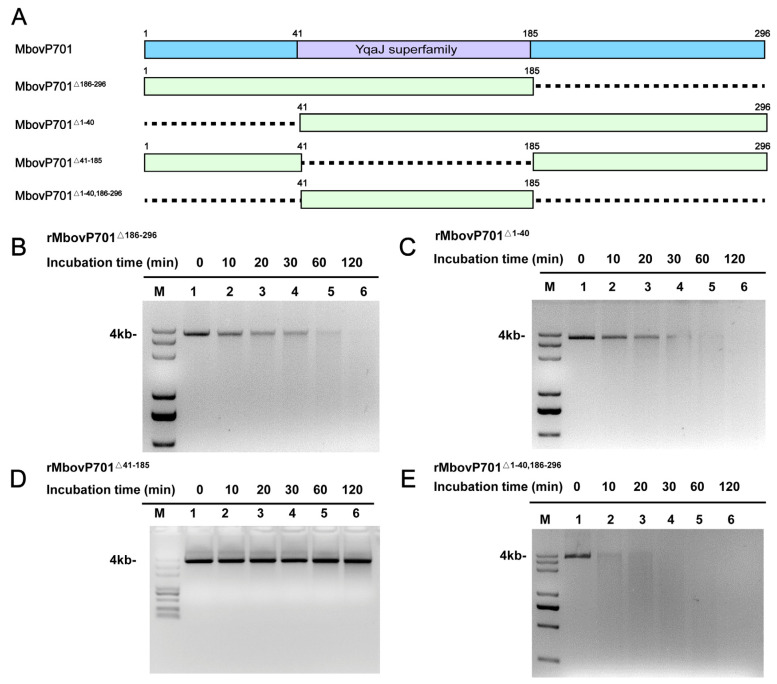
The nuclease activity of the truncated type of rMbovP701. (**A**) A pattern diagram of the truncated region of the MbovP701 protein. (**B**–**E**) 4k dsDNA of pET-30a(+) digested by rMbovP701^Δ186–296^ (**B**), rMbovP701^Δ1–40^ (**C**), rMbovP701^Δ41–185^ (**D**), and rMbovP701^Δ1–40,186–296^ (**E**).

To identify the key enzyme-active regions of MbovP701, we constructed four truncated MbovP701 mutants, including three single-fragment mutations and one double-fragment mutation, and successfully purified them for nuclease activity experiments (Figure 5A and Figure S2). Nuclease activity assays revealed that truncated rMbovP701^Δ186–296^, rMbovP701^Δ1–40^, and rMbovP701^Δ1–40,186–296^ retained their exonuclease function (Figure 5B,C,E). Conversely, dsDNA was not degraded by the truncated rMbovP701^Δ41–185^, which lacks the predicted YqaJ exonuclease domain (Figure 5D). These results unequivocally demonstrate that the aa 41–185 region, the predicted YqaJ domain, is an essential domain for rMbovP701 nuclease activity.

## 4. Discussion

Due to the extremely limited biosynthetic capabilities of mycoplasma, a parasitic lifestyle is adopted in order to obtain the nutritional requirements of this organism from host cells [27,28]. Nucleotide metabolism is a vital process for the survival of mycoplasma, with nucleases playing a pivotal role in the degradation of extracellular DNA (eDNA) and the provision of nucleotide resources [29,30,31]. During pathogen–host interactions, microorganisms frequently encounter eDNA, which can be released from tissues undergoing necrosis, apoptosis, autophagy, and pyroptosis, or actively from living cells as vesicles or lipoprotein complexes [32]. However, to facilitate the absorption and utilization of nucleic acid by mycoplasma, eDNA must be degraded into smaller nucleotide fragments by nucleases. Numerous nucleases in mycoplasma participate in eDNA degradation, such as the MunA of *M. pulmonis* [33] and Mpn491 of *M. pneumoniae* [34] (Substrate: DNA); the MnuA of *M. bovis* [35] (Substrate: dsDNA and plasmid); the MbovNase of *M. bovis* [6] (Substrate: dsDNA, RNA, and plasmid); the MAG_5040 of *M. agalactiae* [36], the MHO_0730 of *M. hominis* [37], the Mpn133 of *M. pneumoniae* [38], the MG_186 of *M. genitalium* [9], and the Mpn597 of *M. hyopneumoniae* [8] et al. (Substrate: ssDNA, dsDNA, RNA, and plasmid). Furthermore, MbovP276, MbovP327, and MbovP328 from *M. bovis* degrade pApA/pGpG to AMP/GMP, with MbovP327 additionally involved in ssRNA degradation [11,12].

Previously, most mycoplasma nucleases were annotated as homologs of the Staphylococcal nuclease (SNase), known for escaping NET entrapment [39]. In this study, we identified MbovP701, a YqaJ-like nuclease in *M. bovis* that is homologous to Lambda Exonuclease and exhibits the capacity to degrade linear dsDNA from the 5′ to the 3′ end. Beyond this, our findings reveal the multifunctional nature of MbovP701, as it can degrade a diverse range of substrates, including dsDNA, ssDNA, RNA, and circular plasmids, potentially reflecting adaptations to *M. bovis*’ pathogenic lifestyle. This versatility may allow *M. bovis* to exploit various nucleic acid sources during infection, enhancing its survival in hostile host environments [1,31]. While further studies are needed to define its substrate specificity and preferences, the broad nuclease activity of MbovP701 suggests it may have versatile roles in *M. bovis* physiology. The observed differences between MbovP701 and Lambda-Exo may be attributed to evolutionary divergence. While Lambda-Exo evolved within a viral context to facilitate homologous recombination and replication in bacteriophages [4,40], MbovP701 has likely adapted for broader enzymatic functions, aligning with *M. bovis*’ metabolic constraints and pathogenic requirements. Additionally, existing studies have shown that the exonucleases of the YqaJ exonucleases rarely function independently; instead, they often form part of a “two-component homologous recombination system” alongside a single-stranded DNA annealing protein [41]. This two-component homology system of virus/phage origin is widely found in prokaryotes, including Lambda Bet/Exo and RecE/T systems [42,43]. In these systems, the exonuclease digests linear dsDNA to generate ssDNA, while the annealing protein facilitates strand pairing and recombination, safeguarding the linearized dsDNA from nonspecific degradation. Based on these observations, we hypothesize that MbovP701 similarly operates in conjunction with an as yet unidentified protein partner in *M. bovis*. This partner likely aids in ssDNA annealing and recombination, enabling homologous recombination repair. Such a mechanism would not only enhance DNA repair efficiency but also promote genetic diversity and adaptability, thereby contributing to the persistence and pathogenicity of *M. bovis*. Further studies could employ co-immunoprecipitation, pull-down assays, and mass spectrometry to identify these partners and elucidate their role in the biology of *M. bovis*. The structural modeling of MbovP701 suggests the presence of flexible regions that enable it to accommodate a wide range of substrates. This flexibility contrasts with the more rigid substrate specificity of Lambda-Exo and likely underpins MbovP701’s multifunctional enzymatic capabilities, allowing it to meet the diverse demands of *M. bovis* during its pathogenic lifecycle.

Although the functional domain of MbovP701 differs from most mycoplasma nucleases, it consistently degrades nucleic acid substrates. The biochemical requirements for the nuclease activity across in different mycoplasma species are similar and rely on the availability of divalent cations, such as Ca^2+^ and Mg^2+^ [44]. Consistent with previous studies, MbovP701 mainly depends on divalent metals Mg^2+^ and Mn^2+^ for its enzymatic function. Interestingly, Mn^2+^ supports the degradation of dsDNA at a much lower optimal concentration (0.2 mmol/L) compared to Mg^2+^ (30 mmol/L). Furthermore, truncation studies of multiple regions within rMbovP701 revealed that the YqaJ-like region is crucial for nuclease activity, although the precise functional sites within this region remain to be elucidated.

The findings of the YqaJ domain nuclease indicate that MbovP701 may serve as a potential target for anti-*M. bovis* infection. The design of small-molecule inhibitors to disrupt the enzyme’s DNA repair functions may result in an increased susceptibility of the bacterium to host immune defenses and antimicrobial treatments. To further explore the multifunctional nature of MbovP701 and its role in *M. bovis* pathogenesis, future research should focus on comparative enzymatic studies to determine its substrate preferences, degradation kinetics, and activity under different physiological conditions, providing insights into whether specific substrates are more efficiently degraded during infection. Detailed structural studies, such as cryo-EM or X-ray crystallography, are essential to identify the regions of MbovP701 that enable its multifunctionality and substrate flexibility, shedding light on the molecular mechanisms underlying its diverse enzymatic capabilities. Investigating how MbovP701’s broad nuclease activity contributes to *M. bovis* immune evasion, nutrient acquisition, and persistence in host environments will be critical to understanding its role in pathogenesis and bacterial survival strategies. Furthermore, a deeper understanding of the role of MbovP701 in the pathogenesis of *M. bovis* may be gained through an investigation of its interaction with host factors. The possibility of MbovP701 engaging with proteins such as helicases or DNA-binding proteins, which may be implicated in the recognition or processing of DNA damage, has yet to be investigated. Beyond its biological significance, MbovP701’s ability to degrade a wide range of nucleic acids presents promising opportunities for its application in molecular biology, particularly in DNA and RNA manipulation technologies. These integrated efforts would deepen our understanding of MbovP701 and enable the development of novel therapeutic and diagnostic strategies against *M. bovis* infections.

## 5. Conclusions

In conclusion, this study confirms that MbovP701 is a thermostable, alkaline exonuclease that depends on the YqaJ domain, exhibiting varying degrees of degradation ability towards dsDNA, ssDNA, RNA, and plasmids, and further demonstrated that the enzyme is indispensable for the growth of *M. bovis*. These findings highlight the significance of Mbov_0701 in the growth and metabolic processes of *M. bovis*, indicating its potential as a target for therapeutic interventions.

## Data Availability

All relevant data are available within the manuscript.

## Supplementary Materials

The following supporting information can be downloaded at: https://www.mdpi.com/article/10.3390/microorganisms12122509/s1, Figure S1: rMbovP701 enzymatic activity on degradation of 4 kb dsDNA under metal ions of K^+^, Na^+^, and Ca^2+^. Figure S2: Expression and purification of rMbovP701 and its truncated proteins; M (kDa) represents protein standards of different molecular masses. Table S1: Oligonucleotide primers used in this study.
