# Supplementary material for: Novel Nuclease MbovP701 with a Yqaj Domain Is Interrelated with the Growth of Mycoplasma bovis
Source: Microorganisms. 2024 Dec 5;12(12):2509. doi: 10.3390/microorganisms12122509 (PMC11678175; doi:10.3390/microorganisms12122509)
Supplement: Supplementary file 1 [file microorganisms-12-02509-s001.zip › Table S1.pdf]

Table S1 Oligo nucleotide primers used in this study

Primer	Oligonucleotide sequence (5′→3′)	Purpose	Product
0701-F1	CATGGAATTCATGGCTAAATACTATAATG GTGT （*Eco*RI）	Mbov_0701 cloning	pET-30a-MbovP0701
0701-R1	CCGTTTAAAGT**CCA**AGAATCATA	Mutagenesis	
0701-F2	AAAAACTATGATTCT**TGG**ACTTT		
0701-R2	ACCCATTTCTTT**CCA**TTTGTC		
0701-F3	TGACAAA**TGG**AAAGAAATGGG		
0701-R3	CCGAAGCTTTTAAAATGATTCAAATGGA AAATC（*Hind*III）	Mbov_0701 cloning	
T7-F	TAATACGACTCACTATAGGG	Verifying MbovP701 nuclease's digestive polarity	712 bp unmodified dsDNA
T7-R	GCTAGTTATTGCTCAGCGG		
T7-PT3-F	TAATACGACTCACTATA$_S$G$_S$G$_S$G		712 bp phosphorothioate-modified dsDNA
T7-PT3-R	GCTAGTTATTGCTCAG$_S$C$_S$G$_S$G		
pet-30a(+)-dsDNA-F	GCTTCCTAATGCAGGAGTCGC	Exploring MbovP701 nuclease's specific substrates	3925 bp dsDNA
pet-30a(+)-dsDNA-R	CGACTGAATCCGGTGAGAATGGC		
ssDNA	ATGAATTTGAGTAATATTCCTAGTAAGA AAAAGACTGTCATAATTTATGGAACAATATG		59 bp ssDNA

Note: The restriction sites for endonuclease *Eco*RI and *Hind*III were shown in underlined; Nucleotide substitutions were shown in bold.
