# Supplementary figures and images for: Novel Nuclease MbovP701 with a Yqaj Domain Is Interrelated with the Growth of Mycoplasma bovis
Source: Microorganisms. 2024 Dec 5;12(12):2509. doi: 10.3390/microorganisms12122509 (PMC11678175; doi:10.3390/microorganisms12122509)

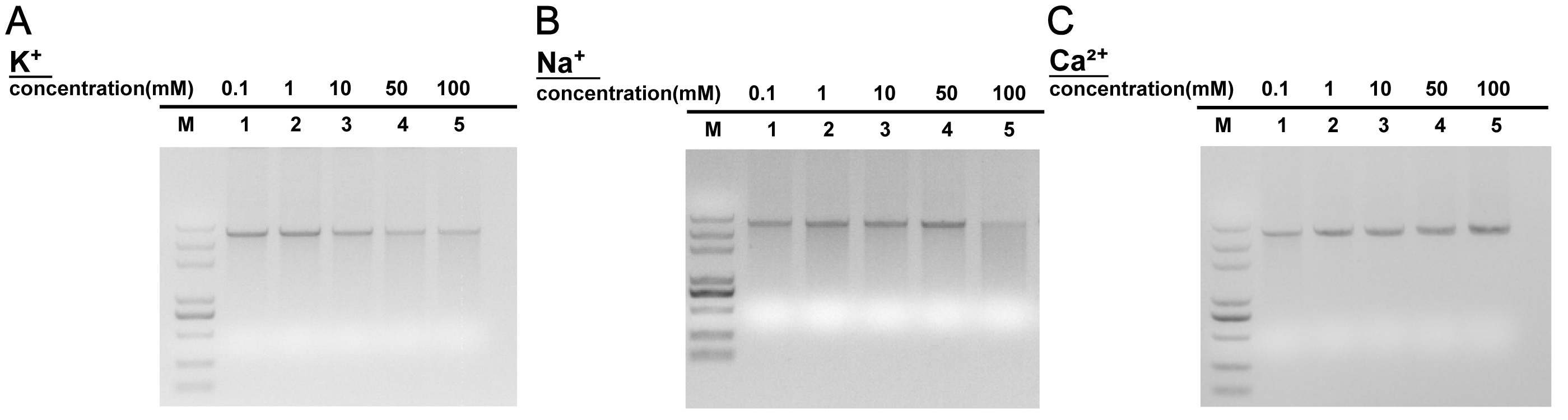

Supplement: Supplementary file 1 [file microorganisms-12-02509-s001.zip › Figure S1.tif]

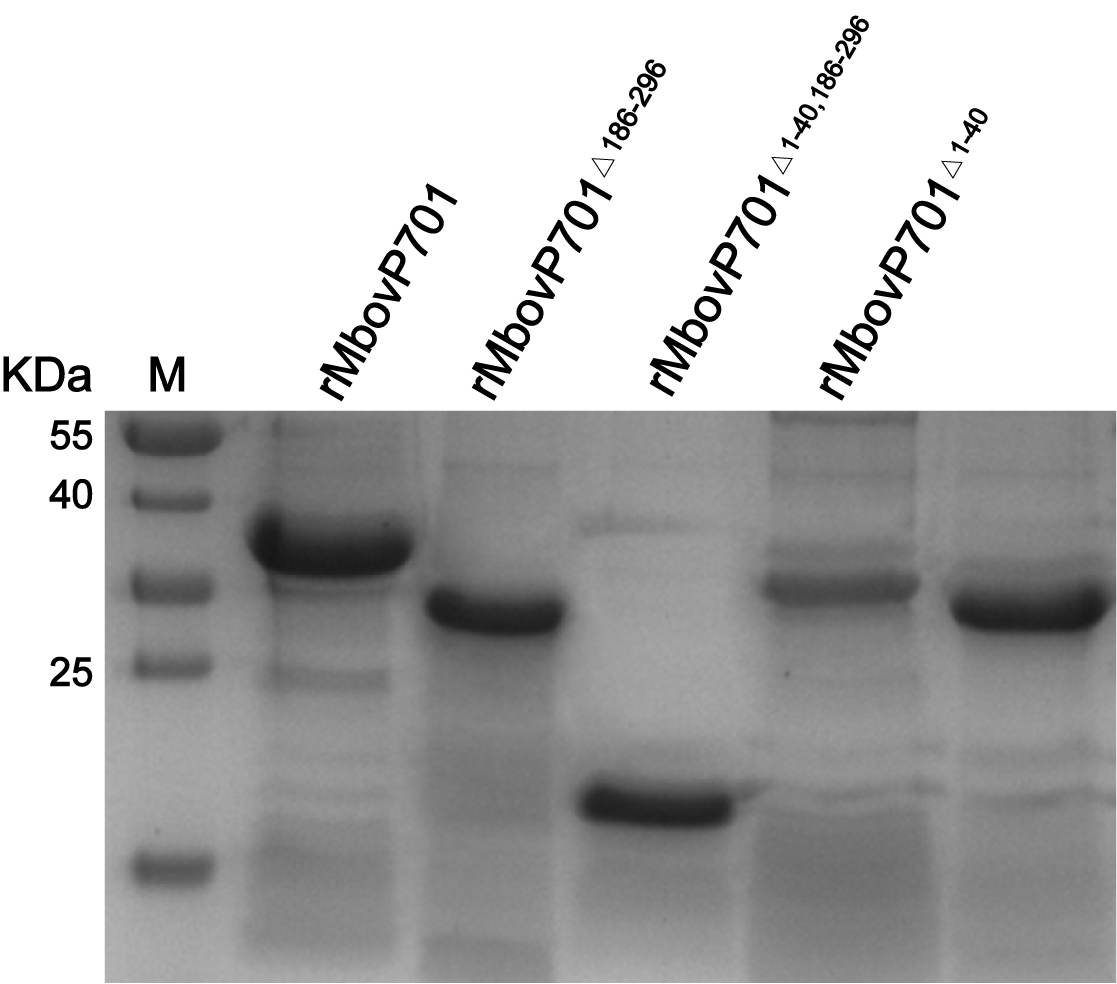

Supplement: Supplementary file 1 [file microorganisms-12-02509-s001.zip › Figure S2.tif]
